# Case report: MRI diagnosis of multifocal epithelioid hemangioendothelioma of the liver

**DOI:** 10.4103/0971-3026.41837

**Published:** 2008-08

**Authors:** Sachit K Verma, Donald G Mitchell, Diane Bergin

**Affiliations:** Department of Radiology, Thomas Jefferson University Hospital, Philadelphia 19107, USA

**Keywords:** Capsular retraction, diagnosis, hepatic epithelioid hemangioendothelioma, liver, MRI

Epitheloid hemangioendothelioma (EHE) is a vascular tumor of intermediate malignancy that arises in soft tissues, liver, lung, bone, and spleen.[1] The hepatic form of multifocal EHE is a rare, less aggressive, slowly progressive tumor, with malignant cells showing dendritic and epitheloid appearances.[2] The tumor is often misdiagnosed because of its nonspecific clinical manifestations and biochemical parameters, the prolonged clinical course, and variable imaging findings. In our patient, characteristic liver contour abnormalities on dynamic contrast-enhanced MRI and histopathological confirmation with appropriate immunohistochemical markers facilitated a correct diagnosis. The only definitive treatment is surgical resection or orthotopic liver transplantation after dedicated radiological and histopathological examination.[3–5]

## Case History

**Figure 1 (A, B):**
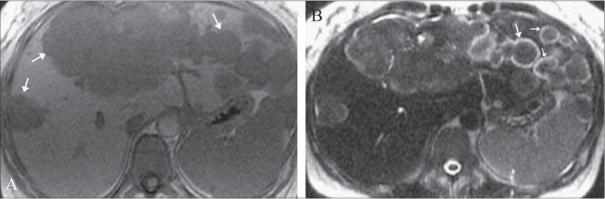
Axial, in-phase spoiled gradient-echo (TR/TE = 120/4.2) image (A) shows multiple, round, moderately low-signal intensity lesions in both lobes of the liver (arrows). Axial, single-shot, fast spin-echo (TE = 180 msec) image (B) reveals the lesions to be isointense with an unusual peripheral rim of T2 hyperintensity (arrows)

**Figure 2 (A-C):**
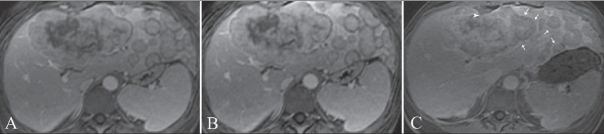
Axial post-gadolinium T1W (120/2.3, 90° flip angle) arterial (A), venous (B), and equilibrium (C) phase images show increased rim enhancement (arrows) in C. Note the capsular retraction (arrowhead)

**Figure 3:**
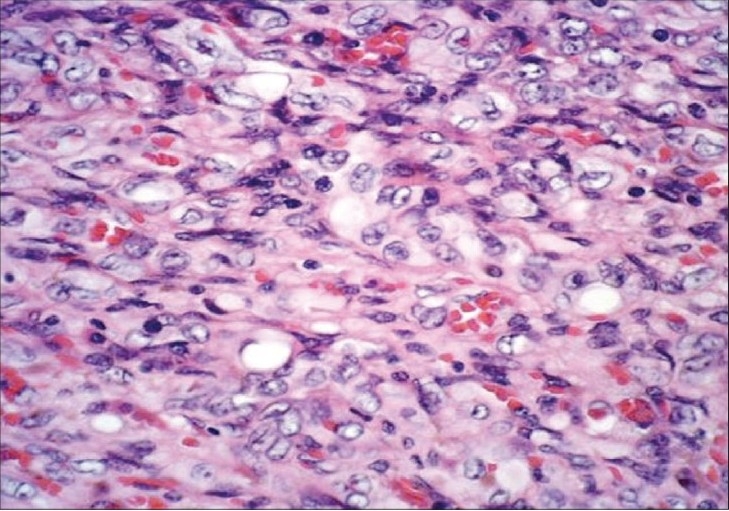
Photomicrograph shows cords of spindle-to-epithelioid cells in a myxoid stroma and prominent vacuoles, some of them containing red blood cells. (H&E, ×50)

A 39-year-old woman presented with right upper quadrant abdominal pain of six months duration. She denied any fever, chills, or malaise. On physical examination, there was painless hepatomegaly. Laboratory values, including serum glutamate oxaloacetate transaminase (SGOT), serum glutamate pyruvate transaminase (SGPT), bilirubin, and alpha-fetoprotein (AFP) levels, were all normal; there was increased alkaline phosphatase (620 U/l) and gamma-glutamyl-transpeptidase (300 U/l) and mild hypoalbuminemia (3 g/dl). She underwent abdominal CT, which revealed conglomerate mass lesions suggestive of metastases in the liver. MRI of the abdomen was then performed on a 1.5-T unit (GE Medical Systems, Milwaukee, USA) using T1W, T2W, and dynamic gadolinium-enhanced sequences. Innumerable small, rounded, predominantly peripheral hepatic lesions, measuring between 1 and 3 cm in size, were identified throughout segments 2 and 3 as well as in the subcapsular right lobe of the liver. These were centrally isointense on T2W and hypointense on T1W images, with an unusual peripheral rim of T2 hyperintensity [Figure 1]. There was moderate enhancement of these lesions except for the hyperintense T2 rim, which showed delayed enhancement [Figure 2]. Some of the lesions showed capsular retraction [Figure 2C]. Multiple, larger, lobulated lesions with similar appearances were seen in segments 4 and 8 [Figure 1B]. Mild right-sided intrahepatic duct dilatation was noted due to mass effect, but there was no obstruction. The patient was subjected to a real-time USG-guided percutaneous liver biopsy. On histological examination, poorly-defined infiltrative tumor was seen, characterized by nests and cords of spindle-to-epithelioid cells embedded in a hyaline, myxoid stroma. The cells showed prominent cytoplasmic vacuoles and intracellular lumina containing red blood cells [Figure 3]. There was positive immunohistochemical staining for the endothelial markers CD31 and CD34. After a definitive diagnosis of multifocal hepatic EHE with no evidence of metastases, liver transplantation was planned for the patient.

## Discussion

Hepatic EHE is a rare clinical entity that was first described by Weiss and Enzinger in 1982.[1] The tumor, often an incidental finding, affects adult women (61%) between 30 and 40 years of age and presents as multiple hepatic nodules.[6] Its etiology is unknown; however, there appears to be an association with regular intake of oral contraceptives, exposure to vinyl chloride, and prior history of trauma to the liver.[4] The tumor shows sclerosis, calcification, and hyalinization in up to 50% of patients.[6] With progression, the multiple hepatic nodular lesions coalesce as they grow at the periphery, resulting in diffuse involvement of the liver.[6]

Among all radiological modalities, MRI provides the best tissue contrast and helps in the diagnosis of EHE. It depicts the internal architecture of hepatic EHE better than CT scan.[7] The MRI findings of coalescent peripheral hepatic masses with capsular retraction are highly suggestive of mutifocal hepatic EHE.[7] The capsular retraction reported in 25-69% of cases is mostly due to a fibrous reaction, which indents the liver capsule.[7,8] In our case, MRI revealed both peripheral as well as infiltrative enhancing hepatic nodules, which were suggestive of an ongoing diffuse process. There was also significant retraction of the liver capsule adjacent to some of the nodules and positive immunohistochemical staining.

The differential diagnosis includes cholangiocarcinoma, fibrolamellar hepatocellular carcinoma, and metastatic carcinoma.[6,7] These can be differentiated from hepatic EHE on the basis of the following: biliary dilatation is usually seen in cholangiocarcinoma; characteristic nodules, cirrhosis, fibrosis, and abnormal liver biochemistry are usually seen in hepatocellular and metastatic carcinoma.[6,9]

Two histological types, nodular and diffuse, have been described. Infiltration into sinusoids and into intrahepatic veins, resulting in luminal narrowing and obliteration with polypoidal projections, are characteristic features of hepatic EHE.[2,3,6] Demonstration of the vascular or endothelial origin of the tumor is critical for diagnosis and requires immunostaining for endothelial markers, including factor VIII-related antigen, CD31, and CD34.[6,10] Surgical resection or orthotopic liver transplantation is considered the treatment of choice, though rarely radiotherapy and chemotherapy may be used in patients with associated metastases.[3–5]

Multifocal hepatic EHE has a better prognosis and better long-term survival than any other hepatic malignancy. The typical MRI features should raise a suspicion in the correct clinical setting and an appropriately guided biopsy will usually give the answer, thus avoiding more complicated diagnostic or therapeutic interventions.
